# Postoperative Changes in Tongue Area and Pharyngeal Airway Space following Mandibular Setback Surgery through Intraoral Vertical Ramus Osteotomy

**DOI:** 10.1155/2021/9923789

**Published:** 2021-07-22

**Authors:** Kwei-Jing Chen, Ying-Ting Chen, Szu-Yu Hsiao, Michael Yuan-Chien Chen

**Affiliations:** ^1^Department of Dentistry and Division of Oral and Maxillofacial Surgery, China Medical University Hospital, Taichung City, Taiwan; ^2^School of Dentistry, China Medical University, Taichung City, Taiwan; ^3^School of Dentistry, CEU Cardenal Herrera University, Valencia, Spain; ^4^Department of Dentistry for Child and Special Needs, Kaohsiung Medical University Hospital, Kaohsiung, Taiwan

## Abstract

**Purpose:**

The aim of this study was to determine changes in the tongue area and pharyngeal airway space (PAS) after intraoral vertical ramus osteotomy (IVRO).

**Materials and Methods:**

Serial lateral cephalograms of 40 patients with mandibular prognathism who underwent IVRO were evaluated before (T1), immediately after (T2), and more than 1 year after (T3) surgery. Paired *t*-tests and Pearson's correlation analysis were used to evaluate the postoperative changes in the mandible, nasopharyngeal airway (NOP), retropalatal pharyngeal airway (RPP), retroglossal pharyngeal airway (RGP), hypopharyngeal airway (HOP), PAS, and tongue area (TA). The null hypothesis states that there are no significant correlations among the extent of mandibular setback and the changes in the TA and PAS after IVRO.

**Results:**

Immediately after the operation (T12), the mandible was set back by 12.6 mm. The NOP, HOP, and PAS were significantly reduced by 35.7 mm^2^, 116 mm^2^, and 185 mm^2^, respectively. The TA was increased by 69.6 mm^2^. The changes in PAS and TA revealed no significant difference between female and male patients at T12, T23, and T13. Moreover, no significant correlations were found among the extent of mandibular setback, TA changes, and PAS changes after IVRO. Thus, the null hypothesis was accepted.

**Conclusions:**

At the final follow-up (T13), no significant change was found in the PAS (including NOP, RPP, RGP, and HOP) and TA. The changes in PAS and TA revealed no significant difference between female and male patients at T12, T23, and T13.

## 1. Introduction

Mandibular prognathism is potentially a genetic disorder and characterized by protrusion of the mandible [1]. It is one of maxillofacial deformities and disfigures the facial appearance. Mandibular prognathism has prevalence as high as 15% in the Asian population and 1% in Caucasians [2, 3]. Surgery to correct mandibular prognathism alters skeletal and soft-tissue components and may cause changes in the tongue and pharyngeal airway space (PAS) [4, 5]. The pharynx is a tubular structure that extends from the cranial base to the sixth cervical spine. From top to bottom, the pharynx can be divided into 3 parts: the nasopharynx, oropharynx, and laryngopharynx (or hypopharynx). In the midsagittal plane, the nasopharynx and oropharynx are set apart by the hard palate. The oropharynx and laryngopharynx are set apart by the epiglottis. The oropharynx is located behind the nasal cavity and oral cavity and above the larynx, trachea, and esophagus [6]. The oropharynx can be further divided into the retropalatal pharynx and retroglossal pharynx set apart by the caudal margin of the soft palate.

The tongue is the most active functional part of the oropharyngeal system and is directly affected by any modification to the orodental environment, especially the mandible [7]. During mandibular setback surgery, the tongue is moved backward, which changes its morphorage. Therefore, the pharynx plays a crucial role in respiration and swallowing. Consequently, the PAS is reduced, which may lead to the onset of obstructive sleep apnea (OSA). OSA is characterized by intermittent and repeated upper-airway collapse during sleep and may lead to breathing cessation. OSA has detrimental effects on nighttime sleep quality and general health due to excessive sleepiness during the day. Riley et al. [8] reported 2 cases of OSA following mandibular setback surgery to correct mandibular prognathism. Therefore, surgeons should be cautious in planning for a large amount of setback in the treatment of mandibular prognathism. The purpose of the present study was to determine the postoperative changes in the tongue area (TA) and PAS following mandibular setback surgery through intraoral vertical ramus osteotomy. The study hypothesized that no significant correlations exist in the postoperative skeletal changes between the TA and PAS after mandibular setback surgery.

## 2. Materials and Methods

### 2.1. Patient Selection

Forty patients with mandibular prognathism who were receiving treatment from the Division of Oral Maxillofacial Surgery at Kaohsiung Medical University Hospital and who fulfilled the following criteria were selected: [1] an Angle Class III malocclusion with mandibular protrusion; [2] no history of trauma or other congenital craniofacial abnormality; [3] no growth of the mandible; and [4] receipt of IVRO alone. This retrospective case study was approved by the Human Investigation Review Committee at the Kaohsiung Medical University Hospital (KMUH-IRB-20140173).

### 2.2. Study Design

**Figure 1:**
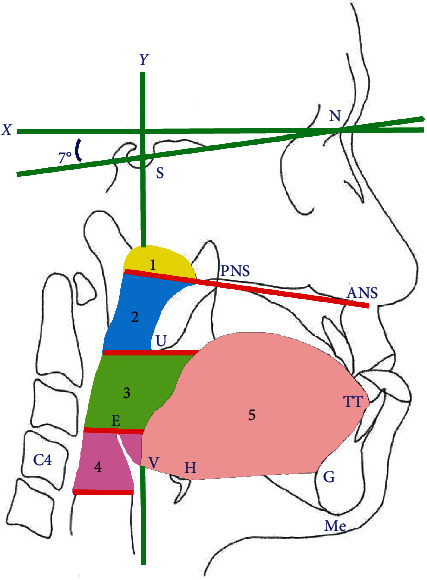
The reference points: S: sella; N: nasion; Me: menton: most inferior point on the mandibular symphysis; ANS: anterior nasal spine; PNS: posterior nasal spine; U: tip of uvula; E: most superior point on the epiglottis; C4: inferoanterior point on the fourth cervical vertebra. The reference lines: SN line; *X*-axis: constructed by drawing a line through the nasion 7° above the SN line; *Y*-axis: constructed by drawing a line through the S perpendicular to the *X*-axis. The pharyngeal airway spaces: (1) NOP: nasopharyngeal airway, yellow color; [2] RPP: retropalatal pharyngeal airway, blue color; [3] RGP: retroglossal pharyngeal airway, green color; [4] HOP: hypopharyngeal airway, pink color; [5] TA: tongue area, flesh color.

Patients were routinely examined using serial cephalograms preoperatively (T1), immediately postoperatively (T2), and more than 1 year postoperatively (T3) to evaluate the postoperative changes in the mandible, TA, and PAS. The reference points and definitions used in this study were as follows (Figure 1): S: sella; N: nasion; Me: menton—the most inferior point on the mandibular symphysis; ANS: anterior nasal spine; PNS: posterior nasal spine; H: the most superior and anterior point of hyoid bone; G: the most prominent point of the mandibular symphyseal posterior border; V: vallecula epiglottica; TT: tongue tip; U: tip of the uvula; E: the most superior point on the epiglottis; and C4: inferoanterior point on the fourth cervical vertebra. The following reference lines were considered: [1] *X*-axis: constructed by drawing a line through the nasion 7° above the SN line and [2] *Y*-axis: constructed by drawing a line through the sella (S) perpendicular to the *X*-axis.

### 2.3. Intervention

The following areas of airway spaces and the tongue were measured: the [1] nasopharyngeal airway (NOP; the area outlined by the roof of pharynx, an extended line of ANS-PNS, and the posterior pharyngeal wall); [2] retropalatal pharyngeal airway (RPP; the area outlined by the inferior border of the nasopharynx, posterior surface of the soft palate, a line parallel to the horizontal plane through point U, and the posterior pharyngeal wall); [3] retroglossal pharyngeal airway (RGP; the area outlined by the inferior border of the retropalatal pharyngeal airway, posterior surface of the tongue, a line parallel to the horizontal plane through point E, and the posterior pharyngeal wall); [4] hypopharyngeal airway (HOP; the area outlined by the inferior border of the retroglossal pharyngeal airway, posterior surface of the tongue and epiglottis, a line parallel to the horizontal plane through point C4, and the posterior pharyngeal wall); [5] PAS: sum of the NOP, RPP, RGP, and HOP; and [6] TA: sagittal tongue above the H-V and H-G lines. The areas of the tongue and PAS were measured using the NIH ImageJ software.

### 2.4. Study Size

To ensure appropriate sample size, at least 35 samples were included to provide a power of 0.8 (alpha = 0.05). The cephalometric landmarks were manually superimposed and identified twice by the author. The intrainvestigator reliability (correlation coefficient: 0.993, *P* < 0.001) was acceptable.

### 2.5. Statistical Analysis

Postoperative changes at the reference points at each time point (T12, T23, and T13) were quantified to estimate statistical parameters, including the mean value and standard deviation. Statistical analysis using the paired *t*-test and Pearson's correlation coefficient was conducted at a confidence level of 95%. The null hypothesis was that no significant correlation exists among the changes in the mandible, TA, and PAS after mandibular setback surgery.

## 3. Results

### 3.1. Participants

The participants comprised 26 female and 14 male patients, with a mean age of 20.5 years (range, 17-34 years). The mean duration of postoperative follow-up was 28.5 months (range, 24-60 months).

### 3.2. Comparisons

**Figure 2:**
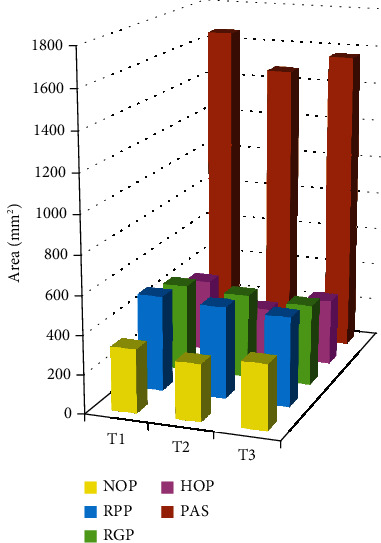
The pharyngeal airway spaces in the T1, T2, and T3 periods. NOP: nasopharyngeal airway (yellow color); RPP: retropalatal pharyngeal airway (blue color); RGP: retroglossal pharyngeal airway (green color); HOP: hypopharyngeal airway (pink color); PAS: pharyngeal airway space (brown color: sum of NOP, RPP, RGP, and HOP).

**Figure 3:**
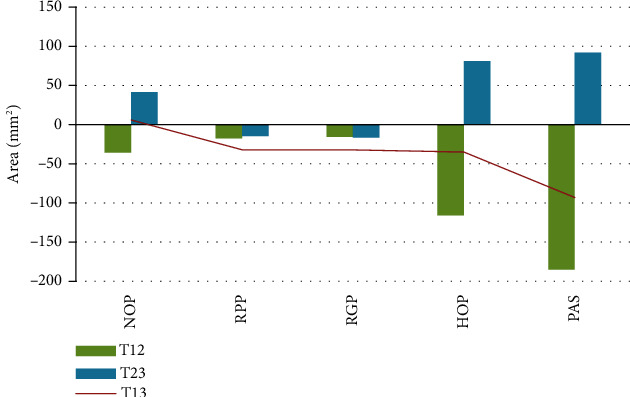
The area changes of pharyngeal airway space in the T12, T23, and T13 measurements. NOP: nasopharyngeal airway; RPP: retropalatal pharyngeal airway; RGP: retroglossal pharyngeal airway; HOP: hypopharyngeal airway; PAS: pharyngeal airway space, sum of NOP, RPP, RGP, and HOP.

As detailed in Table 1, the preoperative areas (Figure 2) were as follows: NOP, 328 mm^2^; RPP, 495 mm^2^; RGP, 452 mm^2^; HOP, 380 mm^2^; PAS, 1655 mm^2^; and TA, 3118 mm^2^. Postoperatively, the Me at T12 and T13 was set back by 12.6 mm (*P* < 0.001) and 11.9 mm, respectively. The Me was significantly more upward (by 1 mm) in the female patients compared with the male patients (downward by 0.5 mm). At T21 (Tables 1 and 2), the NOP was reduced by 35.7 mm^2^ (*P* = 0.004), HOP was reduced 116 mm^2^ (*P* < 0.001), and PAS was reduced by 185 mm^2^ (*P* < 0.001). The changes in the pharyngeal airway spaces (NOP, RPP, RGP, HOP, and PAS) are shown in Figure 3. The TA was increased by 69.6 mm^2^, but the difference was without significance. The PAS and TA were not significantly different between the female and male patients at T12, T23, or T13.

The significant changes at T23 were as follows: NOP increased by 41.6 mm^2^ (*P* = 0.001), HOP increased by 81 mm^2^(*P* = 0.001), and TA reduced by 93.5 mm^2^ (*P* = 0.025). The significant changes at T13 were as follows: Me was moved backward by 11.9 mm (*P* < 0.001), and the PAS reduced by 93 mm^2^ (*P* < 0.001). The decreases in the areas of RPP and RGP were similar at T12, T23, and T13. The areas of the NOP and HOP were decreased at T12 and were restored at T13.

### 3.3. Outcomes

As shown in Tables 3 and 4, horizontal and vertical movements of the Me had no significant correlations with the PAS and TA at T12 and T23. However, at T13, a significant correlation (*r* = 0.409, *P* < 0.01) was noted between horizontal changes in the Me and HOP (Table 5). This result indicates that a large extent of mandibular setback significantly reduced the HOP but had no significant effect on the NOP, RPP, RGP, PAS, or TA. No significant correlation was observed between the TA and PAS at T12, T23, or T13 (Table 6). Moreover, no significant correlations were found among the extent of mandibular setback, TA changes, and PAS changes after IVRO. Therefore, the null hypothesis was supported.

## 4. Discussion

Mandibular setback surgery for the treatment of mandibular prognathism can alter the position of the tongue base, hyoid bone, genioglossus muscle, and geniohyoid muscle, which may further narrow the PAS. In 1995, Deegan [9] claimed that the patency of the pharyngeal airway mainly depends on the effect of the oropharynx muscle. If the pressure exceeds the load of the oropharynx muscle, the pharynx cavity can collapse. Many studies [10–13] have shown that OSA reduces both tonic and phasic activities of the genioglossus, geniohyoid, tensor palatini, levator palatini, palatoglossus, and other respiratory muscles at sleep onset.

Whether the PAS can collapse severely to cause airway complications after mandibular setback surgery remains controversial. Efendiyeva et al. [14] found no significant change in the nasopharyngeal area postoperatively at 5-month follow-up compared with before the operation. Aydemir et al. [15] reported that the nasopharyngeal area was significantly increased by 13.1%. They concluded that the increase in the nasopharyngeal area occurred to compensate for the reduction in the oropharyngeal and hypopharyngeal airway collapse after mandibular setback surgery. In our study, the NOP was significantly reduced by 10.9% (35.7 mm^2^) immediately after the operation. We found that the causes of NOP narrowing were postoperative swelling of the posterior nasopharyngeal wall due to intubation after a larger setback (12.6 mm). Because of the increased extent of setback following increased tissue dissection, more postoperative swelling and edema may have occurred. However, postoperative swelling and edema subsided during follow-up, and the recovery of the NOP area (T3) significantly increased by 12.7% (41.6 mm^2^). Therefore, the NOP nonsignificantly increased by 1.8% (5.9 mm^2^) from before the operation to the 1-year follow-up.

In the literature, Tselnik and Pogrel [5] reported that the immediate postoperation oropharyngeal area (retropalatal and retroglossal pharyngeal area) was significantly increased by 6.1% (70 mm^2^). In our study, the oropharyngeal areas decreased nonsignificantly by 3.5% (33.1 mm^2^). At the 6-month follow-up after mandibular setback surgery, Efendiyeva et al. [14], Jakobsone et al. [16],, and Park et al. [17] reported a nonsignificant decrease. Tselnik and Pogrel [5] reported that the oropharyngeal airway space was significantly decreased by 12.8% (152 mm^2^) in the long-term follow-up. Aydemir et al. [15] found that the upper oropharyngeal area was significantly decreased by 16.6%, but the lower oropharyngeal area was nonsignificantly decreased by 18% after mandibular setback operation. Güven and Saraçoğlu et al. [18] revealed that the area of the oropharyngeal airway space was significantly decreased by 11.6%. In our study, the RPP and RGP areas nonsignificantly decreased by 6.5% (32.1 mm^2^) and 7.1% (32.2 mm^2^), respectively, over a 1-year follow-up.

Immediately after the operation, the HOP of our patients was significantly decreased by 30.5% (116 mm^2^). Fortunately, the HOP significantly increased by 21.3% (81 mm^2^) from T2 to T3 and nonsignificantly decreased by 11.4% (35 mm^2^) over the 1-year follow-up (T13). Enacar et al. [4] reported a significant decrease in the hypopharyngeal airway space following mandibular setback surgery. They suggested that narrowing of the hypopharyngeal airway space due to posterior and inferior movement of the tongue can be permanent. By contrast, Jakobsone et al. [16] found no significant change in the hypopharyngeal airway space at final follow-up. Our results were similar to the report of Jakobsone et al. [16]

Postoperatively, the total PAS in our patients was significantly decreased by 11.2% (185 mm^2^) and 5.6% (93 mm^2^) immediately after surgery and over a 1-year follow-up, respectively. We also found that the HOP was majorly reduced in the total PAS immediately after the operation. During the follow-up period, the HOP also showed a major increase in the total PAS. Moreover, over a 1-year follow-up, the HOP presented a major reduction in the total PAS. Hochban et al. [19] explored the correlation between the extent of setback and change in the PAS and reported that the PAS area was significantly decreased but had no significant correlation with the extent of setback. Tselnik and Pogrel [5] reported a strong correlation between the extent of mandibular setback and the decrease in the PAS area. In our study, no significant correlation was found between the movement of the mandible and alternation of total PAS at T21, T32, and T13. However, the extent of setback had a significant effect on the narrowing of the HOP at T13.

Pae et al. [20] discovered that posterior tongue pressure and genioglossus muscle activity were significantly increased by a change from the upright to supine position in symptom-free controls. Therefore, immediately after mandibular setback surgery, patients may experience pressure from backward bending of the tongue in the supine sleeping position. Otherwise, respiratory distress would occur and potential to onset of OSA. Achilleos et al. [21] analyzed the change in tongue area after mandibular setback surgery and found no significant change at the postoperative 6-month follow-up and a significant increase at the 3-year postoperative follow-up. Jakobsone et al. [16] evaluated changes in the upper airway after bimaxillary correction of Class III malocclusion and found that the tongue length was increased significantly by 4.8 mm and tongue area reduced nonsignificantly by 62 mm^2^. In our study, the tongue area was nonsignificantly increased by 69.6 mm^2^ at T12 and then significantly decreased by 93.5 mm^2^ at T23. Therefore, the tongue area was nonsignificantly decreased by 23.9 mm^2^ at T13.

Tselnik and Pogrel [5] reported that in patients with other risk factors—for example, those who are overweight, with a short neck, or with a large tongue—a mandibular setback procedure can cause predisposal to the development of sleep apnea syndrome. Lowe et al. [22] analyzed the interaction between craniofacial structures and the upper PAS in patients with OSA through linear regression analysis and revealed that a high apnea index was associated with a large tongue volume. In our study, the reduction in RGP was not significantly correlated with the extent of mandibular setback. Many studies have considered OSA to most likely occur at the oropharyngeal (retropalatal and retroglossal) airway space. In a study by Shigeta et al. [23], patients with OSA had a longer soft palate in proportion to their oropharyngeal airway compared with normal controls. Pae et al. [20] emphasized that the vertical and anteroposterior position of the tongue and its relationship to airway size may be more important than the soft palate size in the pathogenesis of OSA. Even with larger extents of setback in our patients, no significant changes in the RPP and RGP were observed between before the operation and the final postoperative follow-up. Moreover, despite the extent of setback in our study being greater than that reported in the literature, no development of respiratory distress was reported in our patients.

Our study has some limitations. First, the mean duration of postoperative follow-up was 28.5 months. Airway complications may occur in patients who gain weight during the long-term follow-up after mandibular setback surgery. Regarding real postoperative PAS, another limitation of the present study was the lack of 3D analysis of the pharyngeal airway. The limitations of a 2D airway analysis are image enlargement issues, distortion, the overlap of bilateral craniofacial structures, and no information on the cross-sectional area. Future research should use 3D images for the assessment of the cross-sectional area and volume of the airway after mandibular setback surgery.

In conclusion, the PAS was not significantly affected by the changes in the mandible and tongue after mandibular setback surgery through IVRO. Although the pharyngeal airway areas were adversely affected after surgery, over the long-term follow-up, recovery and adaptation occurred.

## Figures and Tables

**Table 1:** Student's *t*-test for significance for the menton (Me), pharyngeal airway space (PAS), and tongue area (TA), in the T12, T23, and T13 periods.

Variable	Total patients (*n* = 40)			Female patients (*n* = 26)		Male patients (*n* = 14)		Intergender
	Mean	SD	*P* value	Mean	SD	Mean	SD	Comparison
Me (mm)								
Horizontal change								
T12	-12.6	4.34	<0.001^∗^	-13.1	4.19	-11.6	4.75	0.318
T23	0.6	3.17	0.225	1.0	3.26	-0.1	3.07	0.266
T13	-11.9	4.02	<0.001^∗^	-12.1	4.06	-11.7	4.23	0.797
Vertical change								
T12	0.6	1.85	0.059	0.8	1.95	0.3	1.74	0.413
T23	-0.5	1.80	0.098	-1.0	1.79	0.5	1.51	0.010^∗^
T13	0.1	1.65	0.743	-0.3	1.64	0.7	1.61	0.084
PAS (mm^2^)								
T12	-185.0	299.76	<0.001^∗^	-189.5	243.28	-176.8	394.50	0.913
T23	91.6	341.87	0.098	128.9	296.28	22.2	416.94	0.406
T13	-93.5	282.32	0.043^∗^	-60.6	316.99	-154.5	199.24	0.258
TA (mm^2^)								
T12	69.6	290.04	0.137	94.0	289.00	7.8	290.93	0.378
T23	-93.5	253.94	0.025^∗^	-113.3	234.10	-45.9	291.41	0.464
T13	-23.9	239.49	0.531	-19.3	264.89	-32.4	192.38	0.859

*n*: number of patients; T12: immediate surgical changes; T23: postoperative stability; T13: over 1-year surgical change. ^∗^Significant *P* < 0.05.

**Table 2:** Student's *t*-test for significance for NOP, RPP, RGP, and HOP airways in T12, T23, and T13.

Variable (mm^2^)		Total patients		
		Mean	SD	*P* value
NOP	T12	-35.7	74.51	0.004^∗^
	T23	41.6	76.27	0.001^∗^
	T13	5.9	95.17	0.696
RPP	T12	-17.6	115.03	0.338
	T23	-14.5	101.48	0.372
	T13	-32.1	117.63	0.092
RGP	T12	-15.5	161.43	0.546
	T23	-16.7	135.08	0.440
	T13	-32.2	148.92	0.179
HOP	T12	-116.2	121.98	<0.001^∗^
	T23	81.1	142.68	0.001^∗^
	T13	-35.1	141.77	0.126

T12: immediate surgical changes; T23: postoperative stability; T13: over 1-year surgical change; NOP: nasopharyngeal; RPP: retropalatal pharyngeal; RGP: retroglossal pharyngeal; HOP: hypopharyngeal. ^∗^Significant *P* < 0.05.

**Table 3:** Pearson's correlation coefficient (*r*) test for the tongue area and pharyngeal airway space between menton (Me) in the T12.

Variable	Horizontal Me			Vertical Me		
(mm^2^)	*r*	*P* value		*r*	*P* value	
NOP	0.016	0.921	─	0.011	0.947	─
RPP	-0.217	0.179	─	-0.153	0.345	─
RGP	-0.147	0.365	─	0.092	0.573	─
HOP	-0.146	0.368	─	0.016	0.923	─
PAS	-0.218	0.177	─	-0.001	0.996	─
TA	0.014	0.933	─	0	0.999	─

T12: immediate surgical changes. Significant *P* < 0.05; ─: not significant. NOP: nasopharyngeal; RPP: retropalatal pharyngeal; RGP: retroglossal pharyngeal; HOP: hypopharyngeal; PAS: pharyngeal airway space, sum of NOP, RPP, RGP, and HOP; TA: tongue area.

**Table 4:** Pearson's correlation coefficient (*r*) test for the tongue area and pharyngeal airway space between menton (Me) in the T23.

Variable	Horizontal Me			Vertical Me		
(mm^2^)	*r*	*P* value		*r*	*P* value	
NOP	0.153	0.347	─	0.204	0.206	─
RPP	0.237	0.14	─	0.080	0.625	─
RGP	0.101	0.534	─	0.106	0.514	─
HOP	0.071	0.665	─	0.144	0.374	─
PAS	0.174	0.283	─	0.171	0.29	─
TA	0.011	0.945	─	-0.095	0.558	─

T23: postoperative stability. Significant *P* < 0.05; ─: not significant. NOP: nasopharyngeal; RPP: retropalatal pharyngeal; RGP: retroglossal pharyngeal; HOP: hypopharyngeal; PAS: pharyngeal airway space, sum of NOP, RPP, RGP, and HOP; TA: tongue area.

**Table 5:** Pearson's correlation coefficient (*r*) test for the tongue area and pharyngeal airway space between menton (Me) in the T13.

Variable	Horizontal Me			Vertical Me		
(mm^2^)	*r*	*P* value		*r*	*P* value	
NOP	0.095	0.559	─	0.151	0.353	─
RPP	-0.192	0.236	─	0.217	0.178	─
RGP	-0.017	0.915	─	0.108	0.507	─
HOP	0.409	0.009	^∗^	-0.056	0.733	─
PAS	0.148	0.361	─	0.170	0.293	─
TA	0.138	0.397	─	0.257	0.109	─

T13: over 1-year surgical change. ^∗^Significant *P* < 0.05; ─: not significant. NOP: nasopharyngeal; RPP: retropalatal pharyngeal; RGP: retroglossal pharyngeal; HOP: hypopharyngeal; PAS: pharyngeal airway space, sum of NOP, RPP, RGP, and HOP; TA: tongue area.

**Table 6:** Pearson's coefficient (*r*) test between the tongue area and pharyngeal airway space in the T12, T23, and T13.

Variable	TA T12			TA T23			TA T13		
(mm^2^)	*r*	*P* value		*r*	*P* value		*r*	*P* value	
NOP	-0.043	0.793	─	-0.147	0.365	─	-0.145	0.373	─
RPP	0.009	0.958	─	-0.127	0.434	─	0.090	0.583	─
RGP	-0.115	0.479	─	-0.201	0.214	─	0.106	0.516	─
HOP	-0.089	0.586	─	-0.136	0.404	─	0.120	0.459	─
PAS	-0.106	0.517	─	-0.207	0.201	─	0.105	0.520	─

T12: immediate surgical changes; T23: postoperative stability; T13: over 1-year surgical change. Significant *P* < 0.05; ─: not significant. NOP: nasopharyngeal; RPP: retropalatal pharyngeal; RGP: retroglossal pharyngeal; HOP: hypopharyngeal PAS: pharyngeal airway space, sum of NOP, RPP, RGP, and HOP; TA: tongue area.

## Data Availability

The data used to support the findings of this study are available from the corresponding author upon request.
